# PSE-HMM: genome-wide CNV detection from NGS data using an HMM with Position-Specific Emission probabilities

**DOI:** 10.1186/s12859-016-1296-y

**Published:** 2016-11-03

**Authors:** Seyed Amir Malekpour, Hamid Pezeshk, Mehdi Sadeghi

**Affiliations:** 1 School of Mathematics, Statistics and Computer Science, College of Science, University of Tehran, Tehran, 14155-6455 Iran; 2 School of Biological Sciences, Institute for Research in Fundamental Sciences (IPM), Tehran, Iran; 3 National Institute of Genetic Engineering and Biotechnology, Tehran, Iran

**Keywords:** Next Generation Sequencing (NGS), Hidden Markov Models (HMMs), Expectation Maximization (EM) algorithm, mixture densities, Copy Number Variation (CNV)

## Abstract

**Background:**

Copy Number Variation (CNV) is envisaged to be a major source of large structural variations in the human genome. In recent years, many studies apply Next Generation Sequencing (NGS) data for the CNV detection. However, still there is a necessity to invent more accurate computational tools.

**Results:**

In this study, mate pair NGS data are used for the CNV detection in a Hidden Markov Model (HMM). The proposed HMM has position specific emission probabilities, i.e. a Gaussian mixture distribution. Each component in the Gaussian mixture distribution captures a different type of aberration that is observed in the mate pairs, after being mapped to the reference genome. These aberrations may include any increase (decrease) in the insertion size or change in the direction of mate pairs that are mapped to the reference genome. This HMM with Position-Specific Emission probabilities (PSE-HMM) is utilized for the genome-wide detection of deletions and tandem duplications. The performance of PSE-HMM is evaluated on a simulated dataset and also on a real data of a Yoruban HapMap individual, NA18507.

**Conclusions:**

PSE-HMM is effective in taking observation dependencies into account and reaches a high accuracy in detecting genome-wide CNVs. MATLAB programs are available at http://bs.ipm.ir/softwares/PSE-HMM/.

**Electronic supplementary material:**

The online version of this article (doi:10.1186/s12859-016-1296-y) contains supplementary material, which is available to authorized users.

## Background

Copy Number Variation (CNV) is a major source of the genetic variations and aberrations in the human genome. In CNV, number of copies of a gene or a segment of the genome differs from one person to other. Duplications, deletions and insertions are common types of CNVs that affect roughly 13 % of the human genome. Several clinically relevant CNVs are < 1 kb in size. However, the length of a CNV may get as large as several mega bases [1] e.g. in the HapMap project CNVs of length up to 200 k bp are detected [2].

Most CNVs are germlines which are inherited from the progenitors. But the other prominent source of this variation is somatic and occurs due to the aberrations in the genetic activities such as recombination among homolog chromosomes, during different cycles of the cell division.

Previously, some studies applied hidden Markov models for the genome-wide CNV detection from array-based Comparative Genomic Hybridization (aCGH) data [3–7]. In recent years, development of the Next Generation Sequencing (NGS) has provided an unprecedented opportunity for the study of the genome-wide variations. Most studies that rely on the NGS data use a read depth approach. CNVfinder [8], CNV-seq [9] and BIC-seq [10] compare one sample genome with the reference genome for the CNV detection. On the other hand, CMDS [11], cn. MOPS [12], rSW-seq [13], and CNAseg [14] can take several individuals into account, and predict CNVs based on the information in all samples.

HMM is also applied for modeling NGS read count data [8, 15]. In [8], an HMM with a Poisson emission probability is applied for modeling the observed read counts per genomic segment, after taking the genome-wide variation in GC contents into account. Also, m-HMM uses a Poisson mixture distribution to model read counts for each copy number state [15]. In this way, m-HMM lowers the effect of random errors in the local variations of the read counts.

Due to the high capabilities of the mate pair and split read data in detecting CNVs, in recent years several methods have been using these reads. Some studies applied these mate reads for detecting indels (insertions and deletions) [16–18]. However, besides detecting indels, some methods benefit the attractive feature of mate reads in predicting genome-wide inversions [19–21] and tandem duplications [22–25]. Also, DB2 is introduced for detecting tandem duplication breakpoints [26].

Since mate pair reads have theoretically different potentials in detecting genome-wide CNVs compared to the methods which rely on the read depth, this paper extends the application of HMMs to model variations in the mate pair reads. This novel parametric probabilistic framework enables HMMs to detect genome-wide tandem duplications, besides detecting deletions.

We propose a new HMM which benefit of having Position-Specific Emission probabilities (PSE-HMM) for modeling the length of the genomic regions with deletions (copy loss) and tandem duplications (copy gain). Indeed, a Gaussian mixture density is considered as the emission probabilities in HMM. Each component of this mixture density models a different type of abnormalities that is observed in the insertion size and direction of mate pairs, after being mapped to the reference genome.

A component of the Gaussian mixture density models the increase in the insertion size of the mate pair, after being mapped to the reference genome. This is the case for the genomic regions with deletions. Second component of the Gaussian mixture density models the mate pairs that are mapped to the reference genome in “everted” orientation. This is the case for mate pairs spanning the tandem duplication. Also for the genomic diploid states, a component of the mixture density is applied for emitting those mate pairs with no abnormalities. In PSE-HMM, the position-specific parameter is considered to be the length of a genomic region with copy number variation and this length corresponds to the parameters of the Gaussian mixture density.

The parameters of each density (component) in the Gaussian mixture density are estimated for each genomic segment separately, and on the basis of the mate pairs that are mapped to that segment. However, components’ multipliers are estimated globally, on the basis of the genome-wide mate pair data. Also, Expectation-Maximization (EM) algorithm is applied for estimating the parameters of the HMM emission and transition distributions.

## Methods

**Fig. 1:**
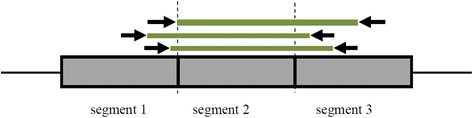
Mate pairs that are taken as the observation for the 2^nd^ genomic segment are shown. A mate pair whose reads are flanking the 2^nd^ segment and its insertion region is spanning the segment, accounts for the observation in the 2^nd^ genomic segment. Other reads that do not satisfy these conditions are discarded

Assume that a sample genome is sequenced via NGS technology and mate pairs are generated. Further, the reference genome is divided into T segments of length L and mate pairs are mapped to the reference genome. In this article, observations for each genomic segment are all those mate pairs whose reads are flanking the segment and their un-sequenced (insertion) regions are spanning the segment, Fig. 1.

Observation vector in the t^th^ genomic segment is shown by ${\mathrm{o}}_{\mathrm{t}}=\left\{{\mathrm{o}}_{\mathrm{t},1},{\mathrm{o}}_{\mathrm{t},2},\dots, {\mathrm{o}}_{\mathrm{t},{\mathrm{n}}_{\mathrm{t}}}\right\}$. Where n_t_ is the number of mate pairs that are mapped to the t^th^segment and the above condition is satisfied for them. Each mate pair’s insertion size is indicated by o_t,i_, where i represents the mate pair index. Observations in genomic segments 1 to T are consequently shown by O = {o_1_, o_2_, …, o_T_}.

Each genomic segment is envisaged to have one of the following states: {homozygous deletion, heterozygous deletion, diploid, tandem duplication}. Indeed, we aim at predicting the copy number of each segment in the sample genome, based on observation vector O. Also, for modeling mate pair data (observation vector O) and predicting the state of each segment, an HMM with inhomogeneous emission probability density is introduced. Indeed, a Gaussian mixture probability density is used to model any aberration in the insertion size and direction of the mate pairs, after mapping to the reference genome.

In the following section, all possible deviations that may occur in the mate pairs’ insertion size and orientation are discussed in details, for each CNV type separately. On the basis of this analysis, a Gaussian mixture density is defined as the emission probability density of the HMM.

### Properties of the HMM states

Each HMM state, i.e. {homozygous deletion, heterozygous deletion, diploid, tandem duplication} has some special properties that are used in our method:

- **Diploid state:** in the human diploid genome each genomic segment appears in two copies, located on a separate homolog chromosome. All mate pairs that pertain to this state have a standard insertion size, after being mapped to the reference genome. Indeed, this insertion size is a feature of the sequencing machine that is used for generating mate pairs from sample genome and it is assumed to be normally distributed with mean μ and variance σ^2^, i.e. N(μ, σ^2^).
- **Homozygous deletion:** in this state both copies of a gene or a genomic segment are deleted. Therefore, all mate pairs that are generated from this state, after being mapped on the reference genome will have an increased insertion size of length μ + deletion size. So, insertion size of these reads will follow a normal distribution of the form N(μ + deletion size, σ^2^).
- **Heterozygous deletion:** this state models the genomic segments for which there is one copy in the sample genome. Therefore, after mapping mate pairs, approximately half of them should have a standard insertion size, i.e. N(μ, σ^2^). However, since one genomic allele is deleted in the sample genome, approximately half of the mate pairs are mapped to the reference genome much further apart than expected with a N(μ + deletion size, σ^2^) distribution.
- **Tandem duplications:** this state models those genomic segments that appear in more than two copies in the sample genome and at least two copies are located one after another and without any space between them, on a homolog chromosome.

**Fig. 2:**
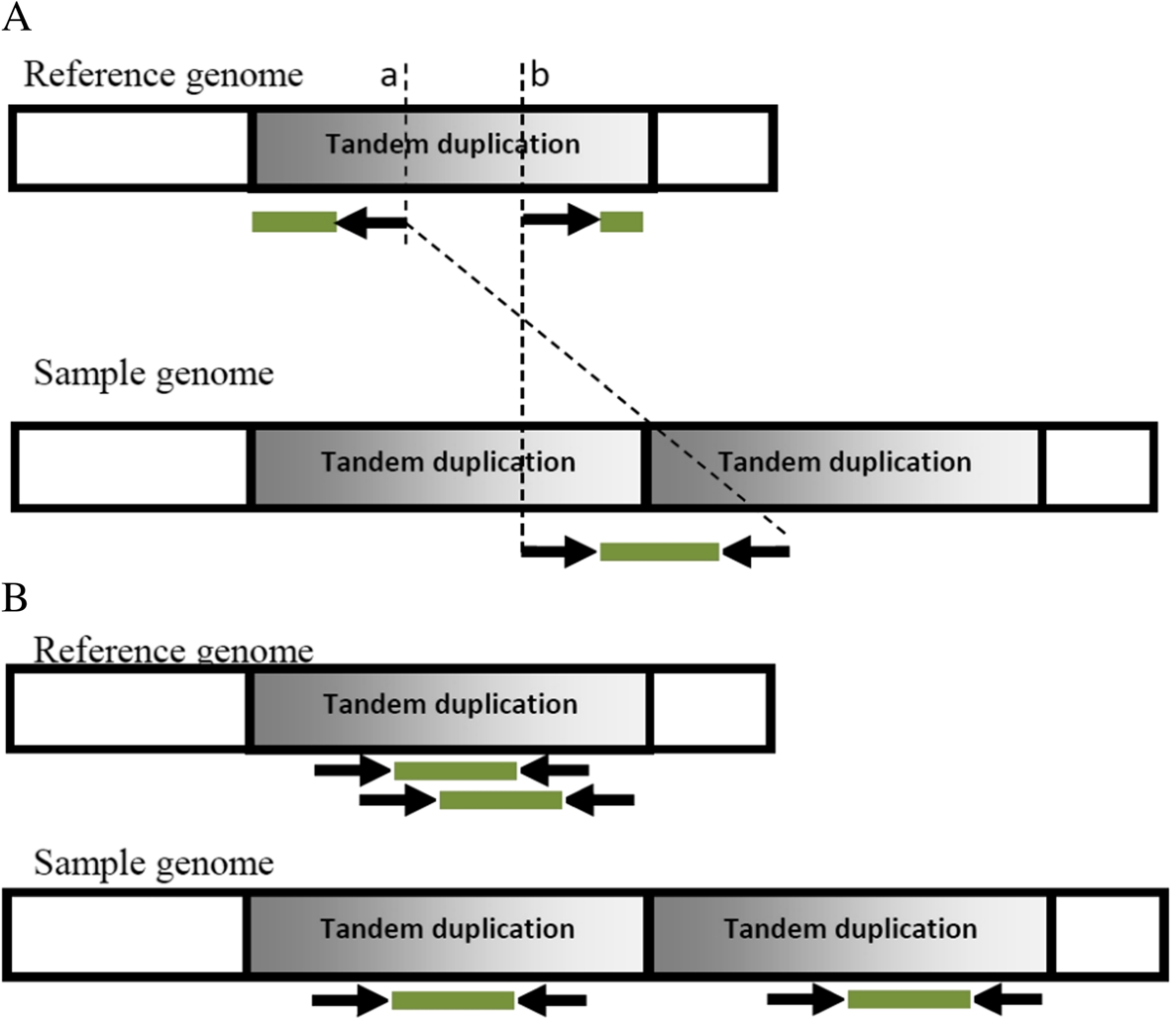
Mate pairs which are generated from a region with tandem duplications, are mapped to reference. Abnormalities in the insertion size and direction of a mate pair depend on whether it is generated from a location around a tandem duplication breakpoint or not. **a** A mate pair spanning the tandem duplication in the sample genome is shown. After mapping to the reference genome, this mate pair encounters a change in direction and abnormality in the insertion size (the distance of point a to b). **b** Two mate pairs that are not located around breakpoint are shown. These pairs will map normally to the reference genome, without any change in the insertion size or direction

Insertion size of a mate pair which is spanning a tandem duplication of length X, after mapping to the reference genome is distributed of the form N(X − μ − 2 ∗ (read length), σ^2^), See Fig. 2a. Clearly, the mean of the insertion size distribution increases linearly with the length of tandem duplication (X). As shown in Fig. 2, these mate pairs after mapping to the reference genome will also have an “everted” orientation.

However, mate pairs that are not generated from locations around the tandem duplications’ breakpoint, after being mapped to the reference genome encounter no change in direction or insertion size, i.e. N(μ, σ^2^), see Fig. 2b.

### HMM structure

**Fig. 3:**
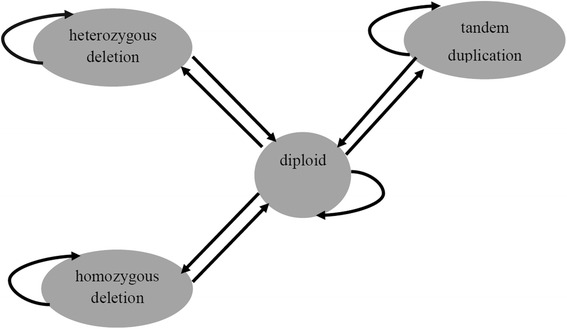
HMM structure, states and transition probabilities are shown. In diploid state each genomic segment has two copies. In heterozygous deletion and homozygous deletion each genomic segment appears in one and no copies, respectively. Duplication state models those genomic segments that have more than two copies in the sample genome, at least one of the tandem duplication type

Each HMM has two major components: transition and emission probabilities. Transition probability is the probability of moving from one state to another in a single step. As shown in Fig. 3, from the diploid state we can reach any other state, i.e. homozygous deletion, heterozygous deletion or a tandem duplication state. From these states we can get back to the diploid state, as well.

Emission probabilities define the probability of emitting the observation sequence from each state. We remind that in the t^th^ genomic segment, observations are insertion size and direction of the pair reads which are indicated by ${\mathrm{o}}_{\mathrm{t}}=\left\{{\mathrm{o}}_{\mathrm{t},1},{\mathrm{o}}_{\mathrm{t},2},\dots, {\mathrm{o}}_{\mathrm{t},{\mathrm{n}}_{\mathrm{t}}}\right\}$, and the corresponding hidden state is indicated by q_t_, where 1 ≤ t ≤ T. Indeed, q_t_ is a member of {homozygous deletion, heterozygous deletion, diploid, tandem duplication}. The probability of emitting observations from different states is summarized in Table 1.

**Table 1:** Expected distribution of the observation in different states

	Distribution		
State	1	2	3
Diploid	*N*(*μ*, *σ* ^2^)	-	-
Heterozygous deletion	*N*(*μ*, *σ* ^2^)	*N*(*μ* + *deletion size*, *σ* ^2^)	-
Homozygous deletion	-	*N*(*μ* + *deletion size*, *σ* ^2^)	-
Tandem duplication	*N*(*μ*, *σ* ^2^)	-	*N*(*X* − *μ* − 2 ∗ *read length*, *σ* ^2^)

In diploid and homozygous states, there is a unimodal distribution for the insertion sizes, while heterozygous deletion and tandem duplication states follow a bimodal insertion size distribution

Based on information in Table 1, in each genomic state the following Gaussian mixture density appears:

$$ \mathrm{f}\left({\mathrm{o}}_{\mathrm{t},\mathrm{k}}\Big|{\mathrm{q}}_{\mathrm{t}}\right)={\upalpha}_{{\mathrm{q}}_{\mathrm{t}},1}{\mathrm{f}}_1\left({\mathrm{o}}_{\mathrm{t},\mathrm{k}}\Big|{\mathrm{q}}_{\mathrm{t}}\right)+{\upalpha}_{{\mathrm{q}}_{\mathrm{t}},2}{\mathrm{f}}_2\left({\mathrm{o}}_{\mathrm{t},\mathrm{k}}\Big|{\mathrm{q}}_{\mathrm{t}}\right)+{\upalpha}_{{\mathrm{q}}_{\mathrm{t}},3}{\mathrm{f}}_3\left({\mathrm{o}}_{\mathrm{t},\mathrm{k}}\Big|{\mathrm{q}}_{\mathrm{t}}\right). $$

It indicates that k^th^ observation in genomic segment t, o_t,k_, 1 ≤ k ≤ n_t_, comes from one of the three indicated densities in Table 1, with a probability of ${\upalpha_{{\mathrm{q}}_{\mathrm{t}},}}_{\mathrm{z}},1\le \mathrm{z}\le 3$. Clearly, $0\le {\upalpha}_{{\mathrm{q}}_{\mathrm{t}},\mathrm{z}}\le 1$ for each q_t_ and ${\displaystyle {\sum}_{\mathrm{z}=1}^3{\upalpha}_{{\mathrm{q}}_{\mathrm{t}},\mathrm{z}}}=1$. Also, o_t,k_ denotes the observed insertion size in a mate pair that is mapped to the reference genome, and f_z_(o_t,k_|q_t_) has the following normal distribution:

$$ {\mathrm{f}}_{\mathrm{z}}\left({\mathrm{o}}_{\mathrm{t},\mathrm{k}}\left|{\mathrm{q}}_{\mathrm{t}}\right.\right)=\frac{1}{\sqrt{2\uppi {\upsigma}_{\mathrm{t}\mathrm{z}}^2}} \exp \left\{\frac{-1}{2{\upsigma}_{\mathrm{t}\mathrm{z}}^2}{\left({\mathrm{o}}_{\mathrm{t},\mathrm{k}}-{\upmu}_{\mathrm{t}\mathrm{z}}\right)}^2\right\}, $$

In which, μ_tz_ and σ_tz_^2^ are the mean and variance of the z^th^ density, in the indicated mixture density. f_1_(o_t,k_|q_t_) models the emission of the insertion size in mate pairs that are mapped to the reference genome with no abnormalities, either in direction or insertion size. The proportion of such mate pairs in the t^th^ genomic segment -which is in the state of q_t_ – is indicated by ${\upalpha}_{{\mathrm{q}}_{\mathrm{t}},1}$. For such mate pairs we assume that μ_t1_ = μ and σ_t1_^2^ = σ^2^. As the sequencing machine is calibrated to generate mate pairs with an insertion that is distributed as N(μ, σ^2^), we show this density by f_1_(. |.).

f_2_(o_t,k_|q_t_) models the insertion size emission in those mate pairs that are mapped to the reference much further apart than expected and has no direction abnormality. ${\upalpha}_{{\mathrm{q}}_{\mathrm{t}},2}$ is the proportion of such mate pairs in the genomic segment. As indicated in Table 1, μ_t2_ = μ + deletion size.

Finally, insertion size in mate pairs with both direction and insertion size abnormalities, is modeled by f_3_(o_t,k_|q_t_), and proportion of such observations in a genomic segment is indicated by ${\upalpha}_{{\mathrm{q}}_{\mathrm{t}},3}$. In genomic segments with tandem duplication state ${\upalpha}_{{\mathrm{q}}_{\mathrm{t}},3}$ is expected to be significantly greater than zero. However, in genomic segments with other states, it is expected to be very close to zero, since some mate pairs may map to the reference genome with direction abnormalities, either due to the sequencing noise or alignment error.

Generally, $\left({\upalpha}_{{\mathrm{q}}_{\mathrm{t}},1},\ {\upalpha}_{{\mathrm{q}}_{\mathrm{t}},2},{\upalpha}_{{\mathrm{q}}_{\mathrm{t}},3}\right)$ for diploid, heterozygous deletion, homozygous deletion, and tandem duplication states are expected to be $\left(1,0,0\right),\left(\frac{1}{2},\frac{1}{2},0\right),\left(0,1,0\right)$, and $\left({\upalpha}_{{\mathrm{q}}_{\mathrm{t}},1},0,1-{\upalpha}_{{\mathrm{q}}_{\mathrm{t}},1}\right)$, respectively.

### Model parameters

There are different parameter sets which have to be estimated:

Transition probabilities: since there are 4 states in the HMM, the probability of transition from state i to state j is denoted by a_ij_, where 1 ≤ i, j ≤ 4 and:

$$ {\mathrm{a}}_{\mathrm{ij}}=\mathrm{p}\left({\mathrm{q}}_{\mathrm{t}}=\mathrm{j}\left|{\mathrm{q}}_{\mathrm{t}-1}=\mathrm{i}\right.\right),\kern0.5em \mathrm{f}\mathrm{o}\mathrm{r}\kern0.5em 2\le \mathrm{t}\le \mathrm{T}. $$

All a_ij_ values are denoted by a 4∗4 matrix.

Emission probabilities: as mentioned before, the probability of emitting o_t,k_, 1 ≤ k ≤ n_t_, in state q_t_ is formulated by the following mixture density:

$$ \begin{array}{cc}\mathrm{f}\left({\mathrm{o}}_{\mathrm{t},\mathrm{k}}|{\mathrm{q}}_{\mathrm{t}}\right)& ={\displaystyle \sum_{\mathrm{z}=1}^3{\upalpha}_{{\mathrm{q}}_{\mathrm{t}},\mathrm{z}}{\mathrm{f}}_{\mathrm{z}}\Big({\mathrm{o}}_{\mathrm{t},\mathrm{k}}\left|{\mathrm{q}}_{\mathrm{t}}\right)},\\ {}& ={\displaystyle \sum_{\mathrm{z}=1}^3{\upalpha}_{{\mathrm{q}}_{\mathrm{t}},\mathrm{z}}\frac{1}{\sqrt{2{\uppi \upsigma}_{\mathrm{t}\mathrm{z}}^2}} \exp \left\{\frac{-1}{2{\upsigma}_{\mathrm{t}\mathrm{z}}^2}{\left({\mathrm{o}}_{\mathrm{t},\mathrm{k}}-{\upmu}_{\mathrm{t}\mathrm{z}}\right)}^2\right\}.}\kern1em \end{array} $$

In which $0\le {\upalpha}_{{\mathrm{q}}_{\mathrm{t}},\mathrm{z}}\le 1$ for 1 ≤ z ≤ 3. The above density depends on genomic position-specific parameters μ_tz_ and σ_tz_^2^ which have to be estimated for each genomic segment, separately. Indeed, the position-specific parameter μ_tz_ determines the length of a genomic CNV region with deletion or tandem duplication and this length is estimated based on information in the mate pairs reads, in the t^th^ genomic segment.

Also, ${\upalpha}_{{\mathrm{q}}_{\mathrm{t}},\mathrm{z}}$ values are global parameters and have to be determined based on the genome-wide mate pair data. These global parameters are state dependent which are the key features in decoding the HMM states.

### Parameter estimation

PSE-HMM applies an Expectation-Maximization (EM) algorithm for the parameter estimation. See Additional file 1: section S.2, for further details.

### Parameter initialization in EM algorithm

T is the genome length which is a fixed value. The segment size (L) can be taken as short as the average insertion size in the clone library. It’s also possible to choose a shorter segment size, as well. However, a shorter segment size results in having more genomic segments which increase the running time of the algorithm. In this study, the segment size is taken to be 150 bp.

The position-specific parameters μ_tz_ and σ_tz_^2^ are initialized either based on the information in the mate pair reads mapped to the genomic segments or based on the prior information from the insertion size distribution in clone library. Transition probabilities are also initialized based on the expected length of the genome-wide CNVs.

Also, to assess and to initialize the proportion of the mate pairs which are mapped to the reference genome with an abnormal orientation $\left({\upalpha}_{{\mathrm{q}}_{\mathrm{t}},3}\right)$, mapping orientations are compared to the expected mate pair orientations in the clone library. The proportion of the mate pairs which are mapped to the reference genome much further apart than expected $\left({\upalpha}_{{\mathrm{q}}_{\mathrm{t}},2}\right)$ is initialized by comparing the mate pair insertion sizes with the insertion size distribution in the clone library.

## Results

PSE-HMM is evaluated on a simulated data set and also on a real data of a Yoruba HapMap individual, NA18507. For data simulation, forward strand of the chromosome 3 of human genome is duplicated. The constructed diploid genome is then altered with deletions (both heterozygous and homozygous) and tandem duplications that are placed randomly. The position, length and the type of each CNV are selected randomly. The generated CNVs are of length 1 kb, 1.5 kb, 2 kb, …, and 5 kb.

Then using MAQ software [27], mate pair reads are simulated from shotgun sequencing of the constructed sample genomes. The insertion size between reads in each mate pair is considered to be normally distributed i.e. N(170, 20^2^). The simulated mate pairs were then mapped to the reference genome. The reference genome is then divided into segments of length 150 nt (Sensitivity of the results to the segment size is studied in Additional file 1: section S.3).

For each genomic segment, we identified mate pairs whose insertion regions are spanning the segment and their reads are flanking the corresponding genomic segment, Fig. 1. The insertion size of these mate pairs are indeed observations that are emitted from the HMM states and are used for the parameter estimation.

Two accuracy measures that are employed are precision and recall (sensitivity) which are TP/(TP + FP) and TP/(TP + FN), respectively. In which, True Positive (TP), False Negative (FN), False Positive (FP) and True Negative (TN). It should be noted that precision is actually 1-FDR (False Discovery Rate) which is of interest. The sensitivity of the results is evaluated for different genomic coverages, i.e. 1×, 5× and 10 × .

Initial values of the parameter vector (α_1_, α_2_, α_3_) and their estimated values after several iterations of the EM algorithm are shown in Table 2, for 10× coverage. For other coverage values, we reached a very similar estimation for this parameter vector.

**Table 2:** Initial parameter vector (α_1_, α_2_, α_3_) and their estimation after several iterations of the EM algorithm

Initial	Estimated
$$ \begin{array}{cc}& \begin{array}{ccc}{\alpha}_1& {\alpha}_2& {\alpha}_3\end{array}\\ {}\begin{array}{c} diploid\\ {}\hfill 1\kern0.5em \mathrm{copy}\hfill \\ {}\hfill 0\kern0.5em \mathrm{copy}\hfill \\ {}\hfill \mathrm{copy}>2\hfill \end{array}& \left[\begin{array}{ccc}\hfill 1\hfill & \hfill 0\hfill & \hfill 0\hfill \\ {}\hfill 0.5\hfill & \hfill 0.5\hfill & \hfill 0\hfill \\ {}\hfill 0\hfill & \hfill 1\hfill & \hfill 0\hfill \\ {}\hfill 0.5\hfill & \hfill 0\hfill & \hfill 0.5\hfill \end{array}\right]\end{array} $$	$$ \begin{array}{cc}& \begin{array}{ccc}{\alpha}_1& {\alpha}_2& {\alpha}_3\end{array}\\ {}\begin{array}{c} diploid\\ {}\hfill 1\kern0.5em \mathrm{copy}\hfill \\ {}\hfill 0\kern0.5em \mathrm{copy}\hfill \\ {}\hfill \mathrm{copy}>2\hfill \end{array}& \left[\begin{array}{ccc}\hfill 0.998\hfill & \hfill \approx 0\hfill & \hfill \approx 0\hfill \\ {}\hfill 0.5\hfill & \hfill 0.5\hfill & \hfill 0\hfill \\ {}\hfill 0\hfill & \hfill 1\hfill & \hfill 0\hfill \\ {}\hfill 0.47\hfill & \hfill 0\hfill & \hfill 0.53\hfill \end{array}\right]\end{array} $$

In Table 3, precision and recall values are calculated for each HMM state i.e. {homozygous deletion, heterozygous deletion, diploid, tandem duplication}, and for 10× depth of coverage.

**Table 3:** PSE-HMM precision and recall are computed for a simulated dataset with 10× depth of coverage

		Real state						
		Heterozygous deletion	Diploid	Homozygous deletion	Tandem duplication	Sum	Precision	Recall
Predicted state	Heterozygous deletions	1,146	660	97	0	1,903	0.60	1.00
	Diploid	2	23,560	2	89	23,653	1.00	0.95
	Homozygous deletions	0	279	1,221	1	1,501	0.81	0.93
	Tandem duplications	3	363	0	2,577	2,943	0.88	0.97
	sum	1,151	24,862	1,320	2,667	30,000		

In columns 3 to 6, predicted state is shown versus the real state of the genomic segments, and number of segments is indicated in the corresponding cell. A total number of 30,000 genomic segments (4.5 million bp) are evaluated in this analysis

Prediction accuracy of PSE-HMM is compared with central CNV detection methods i.e. m-HMM, CNV-seq, Pindel and Delly. As mentioned before, m-HMM and CNV-seq rely on read depth approach and do not discriminate tandem duplications from other types of duplications. However, Pindel and Delly are capable of this, because of using mate pair reads. For comparisons, coverage is allowed to vary from 1× to 10×. Also, to measure the CNV detection uncertainty, the whole simulation study is repeated five times. In each run, precision and recall values are calculated for each CNV state, separately. Then for each method, average and standard deviation of prediction accuracies over five different runs of the whole study are computed and shown in Table 4.

**Table 4:** Precision and recall values of PSE-HMM are compared to m-HMM, Pindel, CNV-seq, and Delly

		Coverage								
		1×			5×			10×		
		Precision mean/std	Recall mean/std	F-measure	Precision mean/std	Recall mean/std	F-measure	Precision mean/std	Recall mean/std	F-measure
Duplications	PSE-HMM	0.91/0.03	0.79/0.02	0.85	0.92/0.02	0.95/0.01	0.93	0.88/0.01	0.97/0.02	0.92
	m-HMM	0.95/0.01	0.21/0.02	0.35	1.00/0.02	0.64/0.02	0.78	1.00/0.01	0.71/0.01	0.83
	Pindel	1.00/0.00	0.11/0.04	0.20	1.00/0.00	0.67/0.03	0.80	1.00/0.01	0.81/0.03	0.90
	CNV-seq	0.55/0.03	0.41/0.03	0.47	0.98/0.00	0.54/0.03	0.70	0.99/0.00	0.57/0.03	0.72
	Delly	1.00/0.00	0.80/0.05	0.89	1.00/0.00	0.99/0.05	0.99	1.00/0.00	1.00/0.00	1.00
Deletions	PSE-HMM (heterozygous)	0.43/0.03	0.37/0.03	0.40	0.54/0.04	0.97/0.01	0.69	0.60/0.02	1.00/0.02	0.75
	PSE-HMM (homozygous)	0.20/0.03	0.92/0.05	0.33	0.73/0.03	0.97/0.02	0.83	0.81/0.02	0.93/0.03	0.87
	PSE-HMM (hetero + homo)^a^	0.31/0.03	0.86/0.02	0.46	0.63/0.02	0.99/0.01	0.77	0.72/0.03	1.00/0.03	0.84
	m-HMM (heterozygous)	0.67/0.02	0.16/0.04	0.25	0.93/0.03	0.88/0.03	0.91	0.93/0.03	0.92/0.02	0.93
	m-HMM (homozygous)	0.95/0.02	0.65/0.02	0.77	0.99/0.02	0.62/0.02	0.77	0.99/0.01	0.62/0.03	0.77
	m-HMM (hetero + homo)^a^	0.93/0.02	0.43/0.03	0.59	0.99/0.01	0.78/0.02	0.87	0.99/0.02	0.80/0.03	0.88
	Pindel	0.93/0.15	0.02/0.01	0.04	0.91/0.03	0.36/0.02	0.52	0.87/0.06	0.45/0.05	0.59
	CNV-seq	0.72/0.05	0.75/0.02	0.73	0.98/0.00	0.91/0.01	0.94	0.98/0.00	0.95/0.01	0.96
	Delly	0.98/0.00	0.32/0.04	0.48	0.99/0.00	0.48/0.03	0.65	0.99/0.00	0.49/0.04	0.66
Diploid	PSE-HMM	0.96/0.00	0.79/0.01	0.87	0.99/0.00	0.93/0.00	0.96	1.00/0.00	0.96/0.00	0.98
	m-HMM	0.87/0.01	1.00/0.01	0.93	0.94/0.00	1.00/0.00	0.97	0.95/0.00	1.00/0.00	0.97
	Pindel	0.82/0.01	1.00/0.00	0.90	0.90/0.01	1.00/0.00	0.95	0.93/0.01	1.00/0.00	0.96
	CNV-seq	0.91/0.00	0.93/0.00	0.92	0.94/0.00	1.00/0.00	0.97	0.95/0.00	1.00/0.00	0.97
	Delly	0.91/0.01	1.00/0.00	0.95	0.94/0.01	1.00/0.00	0.97	0.94/0.01	1.00/0.00	0.97

For each method, the average and standard deviation of the precision (recall) values over five different runs of the whole simulation study are given in each cell. For each state i.e. tandem duplication, deletion and diploid, evaluations are done for three different coverage values i.e. 1×, 5×, and 10×. The implanted CNVs are of length 1 kb, 1.5 kb, 2 kb, 2.5 kb, …, 4.5 kb, and 5 kb

^a^ hetero + homo stands for copy loss

As shown in Table 4, according to F-measure, Pindel and Delly reached very drastic accuracies in detecting genome-wide deletions for all coverages. CNV-seq also reached a very drastic accuracy in predicting duplications. However, PSE-HMM is always ranked among top methods in all states. To have a better understanding of the performance of PSE-HMM in comparison with other state-of-the-art of methods, arithmetic and harmonic means of F-measures are calculated over different HMM states i.e. deletions, duplications and diploid states. As shown in Table 5, PSE-HMM has reached the highest accuracies according to the arithmetic and harmonic means of F-measures, compared to m-HMM, Pindel, Delly and CNV-seq and for coverages of 5×, and 10 × .

**Table 5:** Arithmetic and harmonic means of F-measures

		Coverage		
		1×	5×	10×
Arithmetic mean	PSE-HMM	0.72	**0.89**	**0.91**
	m-HMM	0.62	0.87	0.90
	Pindel	0.38	0.76	0.82
	CNV-seq	0.71	0.87	0.89
	Delly	0.77	0.87	0.87
Harmonic mean	PSE-HMM	0.66	**0.88**	**0.91**
	m-HMM	0.53	0.87	0.89
	Pindel	0.09	0.71	0.78
	CNV-seq	0.66	0.85	0.87
	Delly	0.71	0.84	0.84

For PSE-HMM, m-HMM, Pindel, CNV-seq, and Delly, arithmetic and harmonic means of F-measures are calculated over different HMM states i.e. tandem-duplications, deletions (either heterozygous or homozygous), and genomic diploid states. The highest accuracies are indicated in bold, for coverages of 5× and 10×

In Table 6, precision and recall values of PSE-HMM and other methods are compared for CNVs of length 1 kb, 3 kb, and 5 kb, separately, and for 10× sequencing coverage.

**Table 6:** PSE-HMM is compared to other tools in detecting genome-wide deletions and tandem duplications of size 1 kb, 3 kb, and 5 kb

		CNV length								
		1 kb			3 kb			5 kb		
		Precision mean/std	Recall mean/std	F-measure	Precision mean/std	Recall mean/std	F-measure	Precision mean/std	Recall mean/std	F-measure
Duplications	PSE-HMM	0.88/0.04	0.89/0.03	0.89	0.90/0.03	0.97/0.03	0.93	0.88/0.03	0.98/0.01	0.93
	m-HMM	1.00/0.00	0.71/0.1	0.83	1.00/0.00	0.76/0.04	0.86	1.00/0.00	0.75/0.03	0.86
	Pindel	1.00/0.00	0.95/0.06	0.98	1.00/0.00	0.82/0.11	0.90	1.00/0.00	0.87/0.1	0.93
	CNV-seq	0.99/0.01	0.53/0.06	0.69	0.99/0.00	0.53/0.02	0.69	0.99/0.00	0.59/0.09	0.74
	Delly	1.00/0.00	1.00/0.00	1.00	1.00/0.00	1.00/0.00	1.00	1.00/0.00	1.00/0.00	1.00
Deletions	PSE-HMM (heterozygous)	0.68/0.10	1.00/0.01	0.81	0.35/0.10	1.00/0.00	0.52	0.70/0.08	1.00/0.03	0.82
	PSE-HMM (homozygous)	0.70/0.11	0.89/0.05	0.78	0.77/0.03	0.80/0.00	0.78	0.75/0.03	1.00/0.00	0.86
	PSE-HMM (hetero + homo)^a^	0.69/0.02	0.96/0.02	0.80	0.64/0.02	1.00/0.02	0.78	0.72/0.02	1.00/0.02	0.84
	m-HMM (heterozygous)	0.97/0.03	0.50/0.31	0.66	0.99/0.01	1.00/0.00	0.99	0.99/0.01	1.00/0.01	0.99
	m-HMM (homozygous)	0.99/0.01	1.00/0.00	0.99	0.99/0.01	1.00/0.00	0.99	0.99/0.01	0.60/0.00	0.75
	m-HMM (hetero + homo)^a^	0.98/0.01	0.67/0.03	0.79	0.99/0.01	1.00/0.02	0.99	0.99/0.01	0.78/0.01	0.87
	Pindel	0.98/0.02	0.39/0.08	0.56	0.85/0.10	0.46/0.08	0.59	0.87/0.11	0.45/0.13	0.59
	CNV-seq	0.98/0.00	0.92/0.04	0.95	0.98/0.00	0.96/0.01	0.97	0.98/0.00	0.96/0.02	0.97
	Delly	0.99/0.01	0.47/0.11	0.64	0.99/0.00	0.51/0.04	0.68	0.99/0.01	0.45/0.12	0.62

The average and standard deviation of the precision (recall) values are calculated based on five different repeats of the whole simulation study with 10× sequencing coverage

^a^ hetero + homo stands for copy loss

**Fig. 4:**
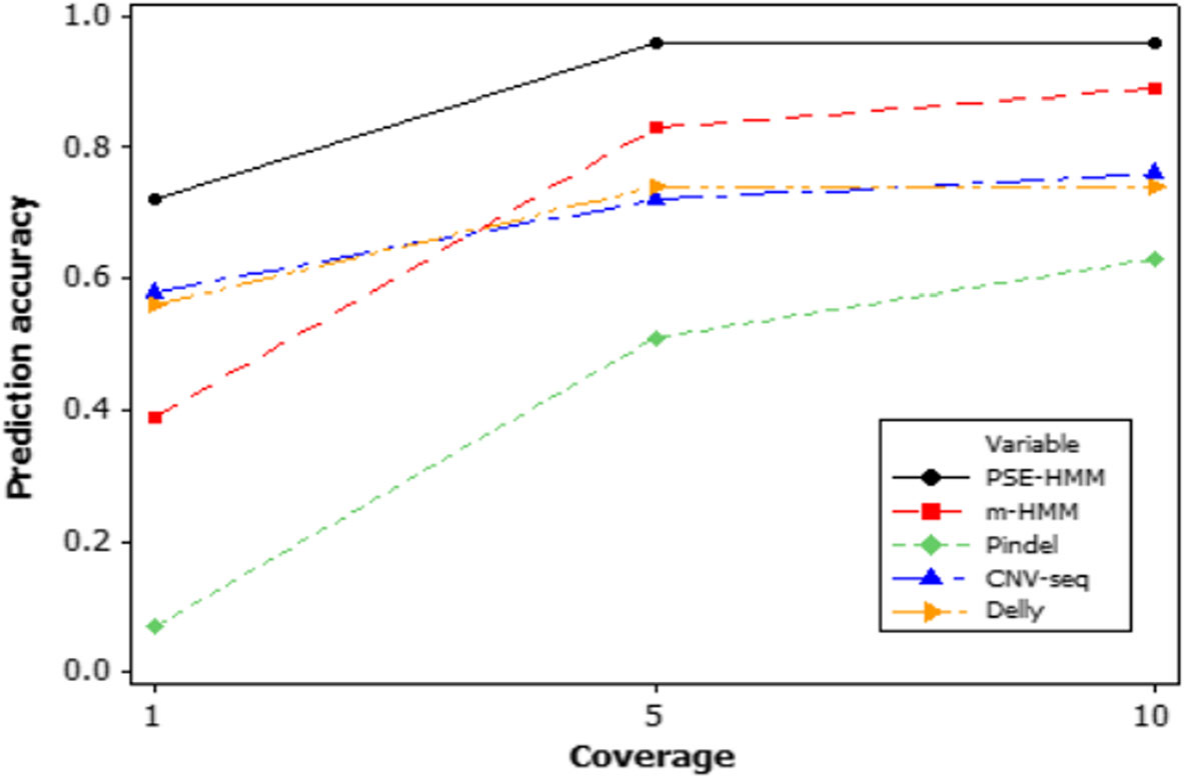
Comparing the overall accuracy of PSE-HMM, m-HMM, CNV-seq, Pindel and Delly in detecting genome-wide CNV regions. Number of nucleotides in CNV regions whose states are correctly predicted is divided by the total length of the genomic CNV regions

PSE-HMM is also compared to other methods, according to overall accuracies in detecting the genome-wide CNV regions (number of nucleotides in CNV regions whose states were correctly predicted are divided by the total length of CNV regions). As shown by Fig. 4, PSE-HMM outperforms m-HMM, CNV-seq, Pindel, and Delly in detecting genome-wide CNV regions, even for low coverage data.

To measure the sensitivity of the result to the genome-wide CNV percentage, we have constructed sample genomes with different CNV percentage. In these sample genomes, the total length of genomic CNV regions over the reference genome length is allowed to vary in the range of 2–30 %, see Additional file 1: section S.4.

### Real data

We applied PSE-HMM for the CNV detection in a male Yoruban HapMap individual from Ibadan Nigeria, NA18507. BAM file of the alignment of the mate pair reads to Build 36 of the human reference genome (hg18) is downloaded from http://ftp.1000genomes.ebi.ac.uk/vol1/ftp/pilot_data/data/NA18507/alignment/. This is a low coverage whole-genome shotgun sequencing data generated by illumina platform. Alignment (.BAM) files are then parsed out using SAMtools (samtools.sourceforge.net) and mate pair reads of low mapping quality (<Q25) are filtered out. After this step, a genome-wide coverage of 1.67× is achieved. In this data, each read is of length 36 bp and the average insertion size is estimated to be 123 bp with a standard deviation of 30 bp.

**Fig. 5:**
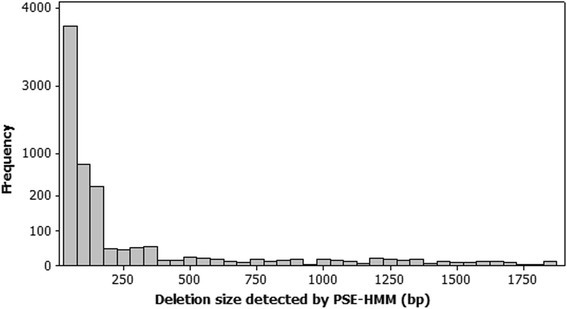
Deletion size distribution for CNVs detected by PSE-HMM, in NA18507. Frequency of the calls decreases exponentially, as deletion size increases

PSE-HMM is applied for detecting deletions and tandem duplications in chromosome 8 of NA18507. PSE-HMM called 5522 CNVs, of which 5447 are deletions of length from 51 to 1871 bp. The other 75 calls are tandem duplications of length in the range of 193 to 46,509 bases. Our calls cover 1.12 % of the studied autosomal chromosome. Also, deletions and tandem duplications cover 0.54 and 0.58 % of the genome, respectively. Distribution of deletion sizes is shown in Fig. 5. Concordant with other studies [25, 28, 29] as deletion size increases, frequency of CNV calls decreases exponentially.

CNVs detected by PSE-HMM are compared with the Database of Genomic Variants (DGV), http://dgv.tcag.ca/dgv/. The DGV contains 8599 identified CNVs in 40 HapMap individuals using aCGH, covering 2.36 % of the genome. As shown by Table 7, 58 % of our calls overlap a call from DGV. Also, from a total number of 1,634,212 bases that are called as a CNV by PSE-HMM, 70 % are also in DGV. In more details, 58 % of the total number of 5447 deletions called by PSE-HMM overlap with a call in DGV (64 % of the bases). Also, 83 % of the 75 tandem duplication calls that are made by PSE-HMM overlap with a call from DGV (75 % of the bases).

**Table 7:** Overlap of CNVs detected by PSE-HMM against DGV is given by calls and bases

	Number of CNV calls	Overlap against DGV (by calls)	Overlap against DGV (by bases)
Deletion	5,447	58 %	64 %
Tandem duplication	75	83 %	75 %
Total	5,522	58 %	70 %

From the statistical point of view, since CNV calls of DGV cover 2.36 % of the genome, a randomly called base by PSE-HMM will also overlap a call from DGV with a probability of 2.36 %. Therefore, overlapping 70 % of the PSE-HMM base calls with DGV is considered statistically significant.

We compared deletions called by PSE-HMM with eight CNV regions of chromosome 8 of NA18507 that are validated to contain a deletion using aCGH methods [29]. The PSE-HMM was able to detect 75 % of the Kidd et al.’s calls (6 out of 8 calls). Overlap of PSE-HMM deletion calls are also investigated against CNVs detected in [30]. For further details see Additional file 1: section S.5.

Moreover, tandem duplications called by PSE-HMM are compared with the study of [31] in which genomic regions with significant intensity difference were identified using aCGH, in a pool of 270 HapMap individual, including NA18507. For this comparison, following the method that was used in [32], PSE-HMM identified 66 % (4 out of 6) of duplications that were made by [31], in NA18507.

## Discussion

The current version of PSE-HMM can be applied for the CNV detection in the diploid genome of human and other organisms, as well. However, it cannot detect CNVs in haploid organisms. Moreover, PSE-HMM reaches accuracies comparable to other state-of-the-art of methods, even using a low coverage data.

Although the current version of the package is limited to whole-genome shotgun sequencing data, further work is in progress to adopt PSE-HMM with the exome or gene panel sequencing data.

The HapMap individual NA18507-used in this study- was sequenced using illumina. However, PSE-HMM may apply for the CNV detection in other platforms, as well. As shown in Additional file 1: section S.6, PSE-HMM will be robust to deviation (skewness) of the insert size distribution from the assumption of normality.

## Conclusion

We proposed PSE-HMM as an HMM with inhomogeneous emission probabilities for the CNV detection from NGS data. PSE-HMM efficiently models the observed deviations in the insertion size and direction of mate pairs, after being mapped to the reference genome. For this purpose PSE-HMM uses a Gaussian mixture density for modeling different types of deviations in the mate pair reads.

Although this article is focused on predicting deletions and tandem duplications, PSE-HMM can be applied for detecting other types of variations, as well.

PSE-HMM outperforms central CNV detection methods i.e. m-HMM, CNV-seq, Pindel and Delly and this indicates that in PSE-HMM, dependencies of observations in consecutive genomic segments are successfully modeled.

## Additional file

Additional file 1: Section S.2 of this additional file provides a detailed description for the parameter estimation in PSE-HMM. In section S.3, the effect of segment size on the performance of the PSE-HMM is investigated. In section S.4, sensitivity of the prediction accuracies to the genome-wide CNV percentage is analyzed. Section S.5 describes the overlap of PSE-HMM’s deletion calls against CNVs which are detected in [30]. Moreover, robustness of PSE-HMM to deviations from the assumption of normality in the insertion size distribution is investigated in section S.6. (DOCX 296 kb)

## Abbreviations

CNV: Copy number variation
EM: Expectation maximization
FDR: False discovery rate
FN: False negative
FP: False positive
HMM: Hidden Markov model
indels: Insertions and deletions
NGS: Next generation sequencing
PSE-HMM: HMM with Position-Specific Emission probabilities
TN: True negative
TP: True positive
