# Supplementary material for: PSE-HMM: genome-wide CNV detection from NGS data using an HMM with Position-Specific Emission probabilities
Source: BMC Bioinformatics. 2016 Nov 3;18:30. doi: 10.1186/s12859-016-1296-y (PMC5445519; doi:10.1186/s12859-016-1296-y)
Supplement: Additional file 1: — Section S.2 of this additional file provides a detailed description for the parameter estimation in PSE-HMM. In section S.3, the effect of segment size on the performance of the PSE-HMM is investigated. In section S.4, sensitivity of the prediction accuracies to the genome-wide CNV percentage is analyzed. Section S.5 describes the overlap of PSE-HMM’s deletion calls against CNVs which are detected in [30]. Moreover, robustness of PSE-HMM to deviations from the assumption of normality in the insertion size distribution is investigated in section S.6. (DOCX 296 kb) [file 12859_2016_1296_MOESM1_ESM.docx]

**PSE-HMM: genome-wide CNV detection from NGS data using an HMM with Position-Specific Emission probabilities**

Seyed Amir Malekpour, Hamid Pezeshk, Mehdi Sadeghi

Additional file 1

**S.1. Overview**

This supplement is organized as follows: section S.2 provides a detailed description for the parameter estimation in PSE-HMM. In section S.3, the effect of segment size on the performance of the PSE-HMM is investigated. In section S.4, sensitivity of the prediction accuracies to the genome-wide CNV percentage is analyzed. Section S.5 describes the overlap of PSE-HMM's deletion calls against CNVs which are detected in ([Mills et al. 2006](#_ENREF_2)). Moreover, robustness of PSE-HMM to deviations from the assumption of normality in the insertion size distribution is investigated in section S.6.

**S.2. Parameter estimation**

For the parameter estimation an Expectation-Maximization (EM) algorithm is applied. In the t^th^ genomic segment with observation vector $o_{t}=\{o_{t,1}, o_{t,2},\ldots,o_{t,n_{t}}\}$, each $o_{t,k}$, $1\leq k\leq n_{t}$, may come from one of the three components of the mixture density, i.e. $f_{z}\left( o_{t,k}|q_{t} \right)$, $1\leq z\leq3$. The index of the component in the mixture density that corresponds to $o_{t,k}$ is denoted by $m_{q_{t}k}$. Therefore, we correspond ${(m}_{q_{t}1},m_{q_{t}2},\ldots,m_{q_{t}n_{t}})$ to the observation vector $o_{t}$. To use EM algorithm, the following Q function is defined:

$Q\left( \theta,\theta^{g} \right)=\sum_{q} \sum_{m} log(P(O,q,m|\theta)P(O,q,m|\theta^{g}))$,

In which $m=\{m_{q_{1}1}, {m_{q_{1}2},\ldots,m_{q_{1}n_{1}},m}_{q_{2}1},\ldots,m_{q_{2}n_{2}},\ldots, m_{q_{T}1}, \ldots,m_{q_{T}n_{T}}\}$, and $\theta$ is the set of all parameter values. $\theta^{g}$ denotes the set of the values of these parameters in the g^th^ iteration of the EM algorithm. For more details see ([Khreich et al. 2012](#_ENREF_1)). In the above equation we sum over all possible values of the m and $q=\{q_{1},q_{2},\ldots,q_{T}\}$.

If $b_{q_{t}}(o_{t})$ denotes the density of emitting $o_{t}$ in state $q_{t}$, the joint density of $O=\{o_{1},o_{2},\ldots,o_{T}\}$ and $q=\{q_{1},q_{2},\ldots,q_{T}\}$ can be written as:

$P\left( O,q | \theta\right)=\pi_{q_{0}}\prod_{t=1}^{T} a_{q_{t-1}q_{t}}b_{q_{t}}(o_{t})$,

In which $\pi_{q_{0}}$ is the probability of starting a sequence of observations $\{o_{1},o_{2},\ldots,o_{T}\}$ in state $q_{0}$and $a_{q_{0}q_{1}}=1$. Therefore,

$$Q\left( \theta,\theta^{g} \right)=\sum_{q} \sum_{m} \log(\pi_{q_{0}})P(O,q,m|\theta^{g})+\sum_{q} \sum_{m} \sum_{t=1}^{T} \log(a_{q_{t-1}q_{t}})P(O,q,m|\theta^{g})+\sum_{q} \sum_{m} \sum_{t=1}^{T} \log(b_{q_{t}}(o_{t}))P(O,q,m|\theta^{g})$$

The first term in the $Q\left( \theta,\theta^{g} \right)$ can be denoted by:

$\sum_{q} \sum_{m} \log(\pi_{q_{0}})P(O,q,m|\theta^{g})=\sum_{q} \log(\pi_{q_{0}})P(O,q|\theta^{g})=\sum_{i=1}^{4} \log(\pi_{i})P(O,q_{0}=i|\theta^{g})$.

Then using the Lagrange multiplier $\gamma$ to introduce the constraint $\sum_{i=1}^{4} \pi_{i}=1$, we have

$$\frac{\partial}{\partial\pi_{i}}\left( \sum_{i=1}^{4} \log(\pi_{i})P(O,q_{0}=i|\theta^{g})+\gamma(\sum_{i=1}^{4} \pi_{i}-1) \right)=0$$

The following formula for updating $\pi_{i}$, in each iteration of the EM algorithm is obtained:

$$\pi_{i}^{g+1}=\frac{P(O,q_{0}=i|\theta^{g})}{P(O|\theta^{g})} .$$

For the second term in $Q\left( \theta,\theta^{g} \right)$ we have:

$$\sum_{q} \sum_{m} \sum_{t=1}^{T} \log(a_{q_{t-1}q_{t}})P(O,q,m|\theta^{g}))=\sum_{i=1}^{4} \sum_{j=1}^{4} \sum_{t=1}^{T} \log(a_{\mathrm{ij}})P(O,q_{t-1}=i,q_{t}=j|\theta^{g}).$$

Again, after introducing a Lagrange multiplier for $\sum_{j=1}^{4} a_{\mathrm{ij}}=1$, and taking derivative with respect to $a_{\mathrm{ij}}$ we obtain:

$$a_{\mathrm{ij}}^{g+1}=\frac{\sum_{t=1}^{T} P(O,q_{t-1}=i,q_{t}=j|\theta^{g})}{\sum_{t=1}^{T} P(O,q_{t-1}=i|\theta^{g})} .$$

Also, for the third term in $Q\left( \theta,\theta^{g} \right)$:

$$\sum_{q} \sum_{m} \sum_{t=1}^{T} \log\left( b_{q_{t}}\left( o_{t} \right) \right)P\left( O,q,m | \theta^{g} \right)=\sum_{q} \sum_{m} \sum_{t=1}^{T} \log\left( \prod_{k=1}^{n_{t}} b_{q_{t}}\left( o_{t,k}, m_{q_{t}k} \right) \right)P\left( O,q,m | \theta^{g} \right),=\sum_{q} \sum_{m} \sum_{t=1}^{T} \sum_{k=1}^{n_{t}} \log b_{q_{t}}\left( o_{t,k}, m_{q_{t}k} \right)P\left( O,q,m | \theta^{g} \right),=\sum_{i=1}^{4} \sum_{z=1}^{3} \sum_{t=1}^{T} \sum_{k=1}^{n_{t}} \log{(\alpha}_{\mathrm{iz}}b_{\mathrm{iz}}\left( o_{t,k} \right)) P(O,q_{t}=i,m_{q_{t}k}=z|\theta^{g}),=\sum_{i=1}^{4} \sum_{z=1}^{3} \sum_{t=1}^{T} \sum_{k=1}^{n_{t}} \log{(\alpha}_{\mathrm{iz}}) P(O,q_{t}=i,m_{q_{t}k}=z|\theta^{g})+\sum_{i=1}^{4} \sum_{z=1}^{3} \sum_{t=1}^{T} \sum_{k=1}^{n_{t}} \log(b_{\mathrm{iz}}\left( o_{t,k} \right)) P(O,q_{t}=i,m_{q_{t}k}=z|\theta^{g}).$$

Since, $\sum_{z} \alpha_{\mathrm{iz}}=1$, by introducing $\lambda$ as the Lagrange multiplier, we get

$$\frac{\partial}{\partial\alpha_{\mathrm{iz}}}\left[ \sum_{i=1}^{4} \sum_{z=1}^{3} \sum_{t=1}^{T} \sum_{k=1}^{n_{t}} \log{(\alpha}_{\mathrm{iz}}) P(O,q_{t}=i,m_{q_{t}k}=z|\theta^{g})+\lambda(\sum_{z} \alpha_{\mathrm{iz}}-1) \right]=0.$$

So,

$$\alpha_{\mathrm{iz}}^{g+1}=\frac{\sum_{t=1}^{T} \sum_{k=1}^{n_{t}} P(q_{t}=i,m_{q_{t}k}=z|O,\theta^{g})}{\sum_{z=1}^{M} \sum_{t=1}^{T} \sum_{k=1}^{n_{t}} P(q_{t}=i,m_{q_{t}k}=z|O,\theta^{g})},$$

In which $P\left( q_{t}=i,m_{q_{t}k}=z | O,\theta^{g} \right)=\gamma_{\mathrm{iz}}(t,k)$ and,

$$\gamma_{\mathrm{iz}}\left( t,k \right)=\gamma_{i}\left( t \right)\frac{\alpha_{\mathrm{iz}}b_{\mathrm{iz}}\left( o_{t,k} \right)}{b_{i}\left( o_{t,k} \right)}=\gamma_{i}\left( t \right)\frac{\alpha_{\mathrm{iz}}b_{\mathrm{iz}}\left( o_{t,k} \right)}{\sum_{z} \alpha_{\mathrm{iz}}b_{\mathrm{iz}}\left( o_{t,k} \right)} .$$

In the above equation, $\gamma_{i}\left( t \right)$ is the posterior probability of the t^th^ genomic segment to be in state i, and is calculated via Forward-Backward (FB) algorithm. Also, $\gamma_{\mathrm{iz}}\left( t,k \right)$ is the joint probability of being in state i in the t^th^ segment for which the k^th^ mate pair comes from mixture component z.

To estimate $\mu_{\mathrm{tz}}$ and $\sigma_{\mathrm{tz}}^{2}$ for $1\leq t\leq T$ and $1\leq z\leq3$, we have:

$$\sum_{i=1}^{4} \sum_{z=1}^{3} \sum_{t=1}^{T} \sum_{k=1}^{n_{t}} \log(b_{\mathrm{iz}}\left( o_{t,k} \right)) P(O,q_{t}=i,m_{q_{t}k}=z|\theta^{g}),=\sum_{i=1}^{4} \sum_{z=1}^{3} \sum_{t=1}^{T} \sum_{k=1}^{n_{t}} (-\ln\sigma_{\mathrm{tz}}-\frac{1}{2\sigma_{\mathrm{tz}}^{2}}\left( o_{t,k}-\mu_{\mathrm{tz}} \right)^{2}) P\left( O,q_{t}=i,m_{q_{t}k}=z | \theta^{g} \right).$$

By taking derivative of the above equation with respect to $\mu_{\mathrm{tz}}$ and setting it equal to zero we get:

$$\sum_{t=1}^{T} \sum_{k=1}^{n_{t}} \frac{1}{\sigma_{\mathrm{tz}}^{2}}{(o}_{t,k}-\mu_{\mathrm{tz}}) P(O,q_{t}=i,m_{q_{t}k}=z|\theta^{g})=0.$$

Then,

$$\sum_{t=1}^{T} \sum_{k=1}^{n_{t}} o_{t,k}P\left( O,q_{t}=i,m_{q_{t}k}=z | \theta^{g} \right)-\mu_{\mathrm{tz}}P(O,q_{t}=i,m_{q_{t}k}=z|\theta^{g})=0.$$

So,

$$\mu_{\mathrm{tz}}^{g+1}=\frac{\sum_{t=1}^{T} \sum_{k=1}^{n_{t}} o_{t,k}P\left( O,q_{t}=i,m_{q_{t}k}=z | \theta^{g} \right)}{\sum_{t=1}^{T} \sum_{k=1}^{n_{t}} P(O,q_{t}=i,m_{q_{t}k}=z|\theta^{g})}.$$

Using the same arguments to estimate $\sigma_{\mathrm{tz}}$:

$$\sum_{t=1}^{T} \sum_{k=1}^{n_{t}} (-\frac{1}{\sigma_{\mathrm{tz}}}+\frac{1}{\sigma_{\mathrm{tz}}^{3}}\left( o_{t,k}-\mu_{\mathrm{tz}} \right)^{2}) P\left( O,q_{t}=i,m_{q_{t}k}=z | \theta^{g} \right)=0.$$

Then, we obtain the following formula for updating $\sigma_{\mathrm{tz}}$

$$\sigma_{\mathrm{tz}}^{g+1}=\left[ \frac{\sum_{t=1}^{T} \sum_{k=1}^{n_{t}} \left( o_{t,k}-\mu_{\mathrm{tz}} \right)^{2}P\left( O,q_{t}=i,m_{q_{t}k}=z | \theta^{g} \right)}{\sum_{t=1}^{T} \sum_{k=1}^{n_{t}} P\left( O,q_{t}=i,m_{q_{t}k}=z | \theta^{g} \right)} \right]^{0.5}.$$

After obtaining the estimated values of the transition and emission probabilities, posterior decoding of HMM states is done with a standard Forward-Backward (FB) algorithm. Indeed, FB calculates the posterior probability of each genomic segment to be in each CNV state, i.e. diploid, heterozygous deletion, homozygous deletion, and tandem duplication.

**S.3. The effect of genomic segment size on the performance of PSE-HMM**

In this section, precision and recall values of PSE-HMM are evaluated for segment size in the range of 135 to 170 bp, for data simulated by MAQ with a normally distributed mate pairs insertion sizes i.e $N(170,{20}^{2})$. It should be noted that considering long genomic segments will decrease the prediction accuracies; as each segment may pertain to the different CNV states. On the other hand, a short segment size results in having more genomic segments which increase the running time of the algorithm.

Therefore, we suggest a sort of confidence interval for the selection of genomic segment size i.e. $[\mu-\sigma,\mu]$, in which $\mu$ and $\sigma$are the average and standard deviation of insertion sizes, respectively. In Table S1, sensitivity of the results is investigated for segment size in the interval of $[\mu-2\sigma,\mu]$, where $\mu=170$ and $\sigma=20$.

**Table S1:** Sensitivity of precision and recall to the segment size. Precision and recall values are evaluated for different segment sizes i.e. 135, 140, 145, 150, 155, 160, and 170 bp and genome-wide coverage of 1×, 5× and 10×. Implanted CNVs are < 1kb in length and mate pair insertion sizes are assumed to be distributed normally i.e. $N({170,20}^{2})$.

|  |  | | segment size | | | | | | | | | | | | | |
| --- | --- | --- | --- | --- | --- | --- | --- | --- | --- | --- | --- | --- | --- | --- | --- | --- |
|  |  |  | 135 | | 140 | | 145 | | 150 | | 155 | | 160 | | 170 | |
|  |  |  | precision | recall | precision | recall | precision | recall | precision | recall | precision | recall | precision | recall | precision | recall |
| Coverage | 1× | heterozygous deletion | 0.46 | 0.36 | 0.43 | 0.32 | 0.43 | 0.29 | 0.39 | 0.24 | 0.32 | 0.16 | 0.30 | 0.13 | 0.30 | 0.01 |
|  |  | diploid | 0.97 | 0.94 | 0.97 | 0.94 | 0.97 | 0.92 | 0.97 | 0.91 | 0.97 | 0.89 | 0.97 | 0.88 | 0.97 | 0.79 |
|  |  | homozygous deletion | 0.30 | 0.91 | 0.27 | 0.92 | 0.25 | 0.92 | 0.22 | 0.92 | 0.18 | 0.92 | 0.17 | 0.92 | 0.10 | 0.92 |
|  |  | tandem duplications | 0.81 | 0.59 | 0.82 | 0.60 | 0.76 | 0.61 | 0.66 | 0.61 | 0.60 | 0.62 | 0.58 | 0.64 | 0.38 | 0.63 |
|  | 5× | heterozygous deletion | 0.55 | 0.98 | 0.62 | 0.93 | 0.54 | 0.96 | 0.44 | 0.88 | 0.51 | 0.82 | 0.47 | 0.72 | 0.39 | 0.27 |
|  |  | diploid | 0.99 | 0.96 | 0.99 | 0.97 | 0.99 | 0.96 | 0.99 | 0.94 | 0.99 | 0.95 | 0.99 | 0.94 | 0.99 | 0.92 |
|  |  | homozygous deletion | 0.57 | 0.88 | 0.59 | 0.95 | 0.56 | 0.95 | 0.50 | 0.97 | 0.45 | 0.99 | 0.39 | 0.98 | 0.22 | 0.98 |
|  |  | tandem duplications | 0.99 | 0.80 | 0.99 | 0.80 | 0.96 | 0.80 | 0.95 | 0.79 | 0.95 | 0.81 | 0.91 | 0.81 | 0.86 | 0.80 |
|  | 10× | heterozygous deletion | 0.51 | 1.00 | 0.51 | 1.00 | 0.51 | 1.00 | 0.52 | 0.99 | 0.54 | 0.96 | 0.52 | 0.91 | 0.45 | 0.61 |
|  |  | diploid | 0.99 | 0.96 | 0.99 | 0.96 | 0.99 | 0.96 | 0.99 | 0.96 | 0.99 | 0.96 | 0.99 | 0.95 | 0.99 | 0.94 |
|  |  | homozygous deletion | 0.58 | 0.81 | 0.56 | 0.83 | 0.56 | 0.84 | 0.56 | 0.89 | 0.52 | 0.95 | 0.50 | 0.96 | 0.34 | 0.99 |
|  |  | tandem duplications | 0.99 | 0.82 | 0.98 | 0.80 | 0.98 | 0.82 | 0.97 | 0.81 | 0.97 | 0.83 | 0.95 | 0.82 | 0.92 | 0.81 |

As shown by Table S1, choosing a reasonable segment size does not affect precision and recall values for the genome-wide CNV states (heterozygous deletion, homozygous deletion, tandem duplications) and diploid state, as well.

**S.4. Sensitivity of the results to the genome-wide CNV percentage**

To measure the sensitivity of results to the genome-wide CNV percentage, we have constructed sample genomes with different CNV percentages. Indeed, the total length of the genomic CNV regions over the reference genome length is allowed to vary in the range of 2%-30%, i.e. 2%, 4%, 6%, 8%, 10%, 12%, 13%, 15%, 18%, 20%, and 30%. Then, the sensitivity of the results is evaluated for sample genomes with various CNV percentages. It should be added that, in the human genome the average CNV percentage is expected to be around 13%. However, it may vary from one individual to other. Overall prediction accuracies for the genomic regions with CNVs is shown in Figure S1. As indicated in this Figure, accuracy in detecting CNV regions is not affected by the genome-wide CNV percentage.


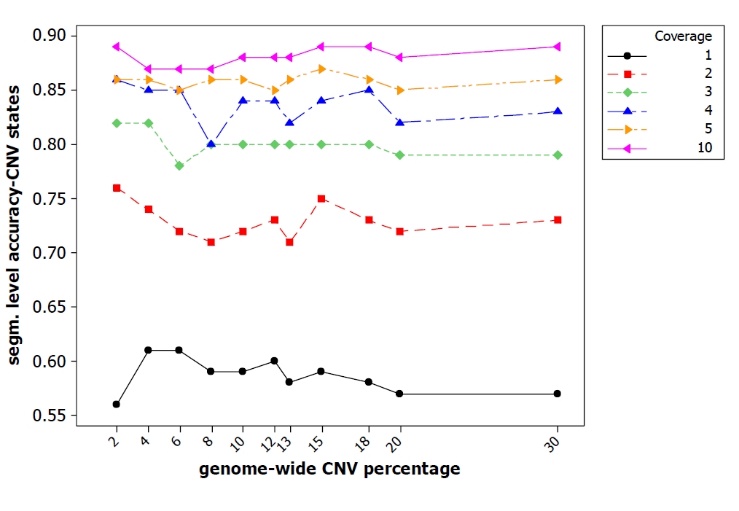


### Fig. S1. Prediction accuracies are evaluated vs. genome-wide CNV percentage, for CNVs < 1kb in length. The CNV percentage of the genome is plotted against the accuracy in predicting the genomic CNV regions (number of genomic segments with CNVs whose states were correctly predicted is divided by the total number of genomic CNV segments).

**S.5. Performance of PSE-HMM in real data**

Distribution of tandem duplication size – for PSE-HMM calls in Yoruban HapMap individual NA18507- is shown in Figure S2.


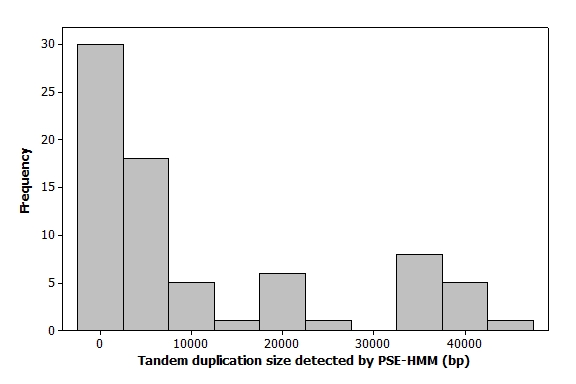


**Fig. S2:** Distribution of tandem duplication size for calls that are made by PSE-HMM. 75 tandem duplications are called with the length in the interval of 193 to 46,509.

PSE-HMM deletion calls are also compared with CNV calls of ([Mills et al. 2006](#_ENREF_2)). In Figure S3, size of PSE-HMM deletion calls are plotted against the size of overlapping deletions in Mills et al..

| A. |
| --- |
|  |
| B. |
| 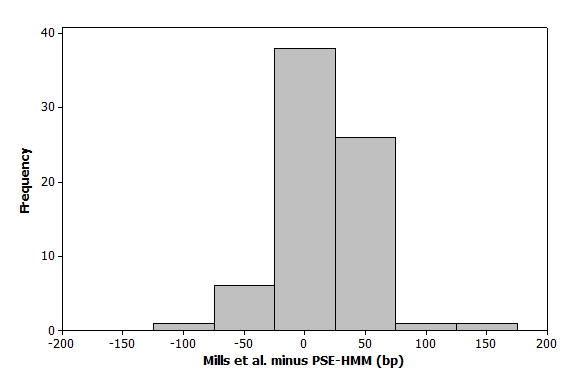 |

**Fig. S3:** Size of overlapping deletions between PSE-HMM and Mills data are compared. A. Scatter plot for the size of overlapping deletions. A Pearson correlation coefficient of 0.92 is observed between the size of overlapping deletions (R^2^=0.84). B. Histogram of the difference between the known deletion sizes and PSE-HMM predictions.

**S.6. Robustness of PSE-HMM to the non-Gaussian insertion size distribution**

In this section robustness of PSE-HMM to the non-Gaussian insertion size distribution is tested. For this purpose, mate pair insertion sizes are generated from distributions that deviate from the Gaussian distributions, with different degree of skewness. For generating insertion sizes a skew normal distribution is used. This continuous distribution generalizes the Gaussian distribution to allow for non-zero skewness. The skew normal probability distribution with location $\mu$, scale $\sigma$ and the parameter $\alpha$ is shown by:

$$f\left( x \right)=\frac{2}{\sigma}\varphi\left( \frac{x-\mu}{\sigma} \right)\Phi\left( \alpha\left( \frac{x-\mu}{\sigma} \right) \right) \sigma>0, \mu and \alpha\epsilon R$$

where, $\varphi$ denotes the standard Gaussian probability distribution with the cumulative distribution function $\Phi$:

$$\Phi\left( y \right)=\int_{-\infty}^{y} \varphi(t)dt$$

The distribution is right skewed if $\alpha>0$ and is left skewed if $\alpha<0$. In Figure S4, the skew normal distribution for different values of parameter $\alpha$ is shown.

|  |
| --- |
| 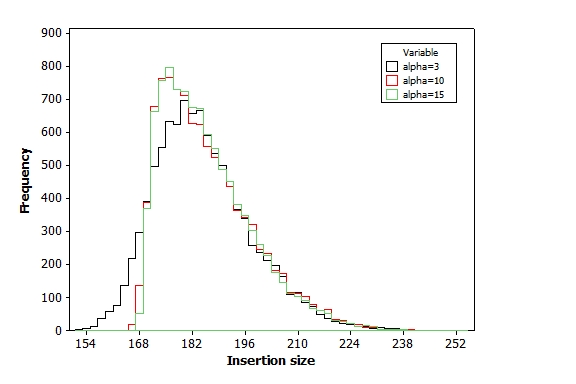 |
|  |
| 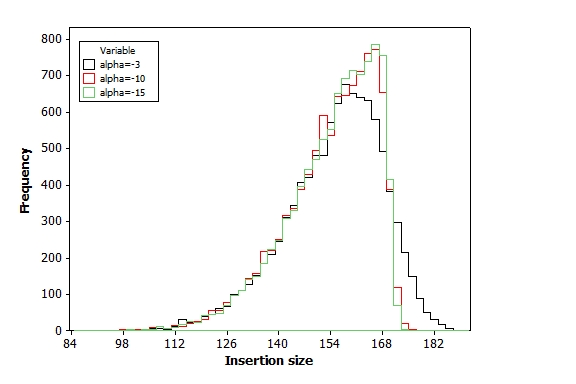 |
|  |

**Fig. S4:** Histogram of skew normal distribution for different values of the parameter alpha, $\mu=170$, $\sigma=20$. 10000 samples are generated for each parameter set. A. Skew normal distribution for $\alpha$ =3,10,15, distribution is right skewed for these parameters. B. Skew normal distribution for $\alpha$ =-3,-10,-15, for which skewness is to the left.

In Table S2, precision, recall and overall accuracy of PSE-HMM are evaluated for different values of skewness in the insertion size distribution. As discussed above, insertion sizes are generated from the skew normal distribution with a parameter $\alpha$ which controls the skewness (and deviation from the Gaussian distribution), in the insertion size distribution. After generating insertion sizes from the skew normal distribution, they are modeled in PSE-HMM via a Gaussian mixture distribution. In this way the robustness of PSE-HMM is measured, for different degrees of deviations in the insertion size distribution.

|  |  | | alpha | | | | | | | | | | | | | |
| --- | --- | --- | --- | --- | --- | --- | --- | --- | --- | --- | --- | --- | --- | --- | --- | --- |
|  |  |  | -15 | | -10 | | -3 | | 0 | | 3 | | 10 | | 15 | |
|  |  |  | precision | recall | precision | recall | precision | recall | precision | recall | precision | recall | precision | recall | precision | recall |
| Coverage | 1× | heterozygous deletion | 0.44 | 0.30 | 0.41 | 0.32 | 0.40 | 0.32 | 0.39 | 0.24 | 0.44 | 0.25 | 0.40 | 0.24 | 0.42 | 0.24 |
|  |  | diploid | 0.97 | 0.92 | 0.97 | 0.92 | 0.97 | 0.92 | 0.97 | 0.91 | 0.97 | 0.91 | 0.97 | 0.91 | 0.97 | 0.91 |
|  |  | homozygous deletion | 0.25 | 0.91 | 0.25 | 0.91 | 0.25 | 0.91 | 0.22 | 0.92 | 0.24 | 0.91 | 0.23 | 0.91 | 0.23 | 0.91 |
|  |  | tandem duplications | 0.66 | 0.61 | 0.66 | 0.61 | 0.66 | 0.61 | 0.66 | 0.61 | 0.66 | 0.61 | 0.66 | 0.61 | 0.66 | 0.61 |
|  | 5× | heterozygous deletion | 0.58 | 0.93 | 0.58 | 0.91 | 0.58 | 0.95 | 0.44 | 0.88 | 0.57 | 0.94 | 0.57 | 0.90 | 0.58 | 0.90 |
|  |  | diploid | 0.99 | 0.96 | 0.99 | 0.96 | 0.99 | 0.96 | 0.99 | 0.94 | 0.99 | 0.96 | 0.99 | 0.96 | 0.99 | 0.96 |
|  |  | homozygous deletion | 0.57 | 0.98 | 0.56 | 0.97 | 0.58 | 0.98 | 0.50 | 0.97 | 0.57 | 0.98 | 0.56 | 0.99 | 0.55 | 0.98 |
|  |  | tandem duplications | 0.94 | 0.79 | 0.94 | 0.79 | 0.94 | 0.79 | 0.95 | 0.79 | 0.94 | 0.79 | 0.94 | 0.79 | 0.94 | 0.79 |
|  | 10× | heterozygous deletion | 0.59 | 1.00 | 0.59 | 1.00 | 0.59 | 1.00 | 0.52 | 0.99 | 0.59 | 0.99 | 0.57 | 0.99 | 0.58 | 0.99 |
|  |  | diploid | 0.99 | 0.96 | 0.99 | 0.96 | 0.99 | 0.96 | 0.99 | 0.96 | 0.99 | 0.96 | 0.99 | 0.96 | 0.99 | 0.96 |
|  |  | homozygous deletion | 0.61 | 0.94 | 0.61 | 0.94 | 0.61 | 0.94 | 0.56 | 0.89 | 0.60 | 0.94 | 0.60 | 0.92 | 0.60 | 0.94 |
|  |  | tandem duplications | 0.96 | 0.81 | 0.96 | 0.81 | 0.96 | 0.81 | 0.97 | 0.81 | 0.96 | 0.81 | 0.96 | 0.81 | 0.96 | 0.81 |

**Table S2:** Precision and recall values are evaluated for different skewness degrees in the distribution of insertion sizes. Insertion sizes are distributed with a skew normal distribution whose skewness is controlled by parameter $\alpha$. These insertion sizes were consequently modeled by PSE-HMM using a Gaussian mixture distribution. For CNVs < 1kb in length, precision and recall values are evaluated for $\alpha$ = -15, -10, -3, 0, 3, 10, and 15 and genome-wide coverage of 1×, 5× and 10×.

As indicated in Table S2, PSE-HMM is robust to various degrees of deviation in the distribution of insertion sizes, from the assumption of normality. Even for the cases in which insertion sizes are harshly skewed, when data are modeled using a Gaussian mixture distribution in PSE-HMM we observe no change in prediction accuracies. The same result is achieved with an increased value of $\sigma$ (standard deviation of insertion size) e.g. using $\sigma=30, 40$.

This can be explained by the CNV size which is long enough to make a considerable shift in the average of insertion size distribution. Therefore, different insertion size classes (with different locations and averages) will yet locate much further apart, even when having harsh deviations from the assumption of normality, in the distribution of insertion size. Indeed, a rough estimation of the average insertion size works fine for the CNV detection, when a skewed distribution is modeled by a Gaussian distribution.

**S.7. Additional references**

Khreich W, Granger E, Miri A, Sabourin R. 2012. A survey of techniques for incremental learning of HMM parameters. *Information Sciences* **197**: 105-130.

Mills RE, Luttig CT, Larkins CE, Beauchamp A, Tsui C, Pittard WS, Devine SE. 2006. An initial map of insertion and deletion (INDEL) variation in the human genome. *Genome research* **16**(9): 1182-1190.
